# Supplementary material for: Learning curve and surgical outcome of robotic assisted colorectal surgery with ERAS program
Source: Sci Rep. 2022 Nov 29;12:20566. doi: 10.1038/s41598-022-24665-w (PMC9709162; doi:10.1038/s41598-022-24665-w)
Supplement: Supplementary file 1 — Supplementary Information 1. [file 41598_2022_24665_MOESM1_ESM.zip › LSC Raw data-Ñ[▒K/061/H_ñw╝╨░O▒Kñσ.pdf]

正式摘要(電子簽章：0000018429966?)

W55 / W55 ☐英文標題(注意:無英文姓名-請補病人基本檔!) ☐【讀取】憑證

臺中榮民總醫院

出院病歷摘要

轉入號： 轉出號：

[redacted]

(8)流水編號	
(9)入院日期	109/01/09 (2020) 直腸外科 W55 - 263
(10)轉科日期	
(11)出院日期	109/01/18 (2020) 住院天數計 9 日

診斷

- 入院:Adenocarcinoma, moderately differentiated, of rectum, 2-8 cm FAV, w/ suspicious perirectal tissue invasion, and small perirectal LNs, cT3N1M0, preCCRT CRP 1.884 mg/dL /p Oxa + Oral Ufur 1# TID + LV since 2017-08-09 /p CCRT until 2017-09-14, he preferred watchful waiting, w/ residual invasive adenocarcinoma noted in 2018-01 re-biopsy specimen, /p peranal excision on 2018-03-25, 2018-04-17, 2018-07-02 with residual tumor
- 出院:Adenocarcinoma, moderately differentiated, of rectum, 2-8 cm FAV, w/ suspicious perirectal tissue invasion, and small perirectal LNs, cT3N1M0, preCCRT CRP 1.884 mg/dL /p Oxa + Oral Ufur 1# TID + LV since 2017-08-09 /p CCRT until 2017-09-14, he preferred watchful waiting, w/ residual invasive adenocarcinoma noted in 2018-01 re-biopsy specimen, /p peranal excision on 2018-03-25, 2018-04-17, 2018-07-02 with residual tumor s/p LAR with loop ileostomy on 2020/01/10

主訴

- residual rectal tumor

病史

- This is a 64 year old male patient with history of

  1.Adenocarcinoma, moderately differentiated, of rectum, 2-8 cm FAV, w/ suspicious perirectal tissue invasion, and small perirectal LNs, cT3N1M0, preCCRT CRP 1.884 mg/dL /p Oxa + Oral Ufur 1# TID + LV since 2017-08-09 /p CCRT until 2017-09-14, he preferred watchful waiting, w/ residual invasive adenocarcinoma noted in 2018-01 re-biopsy specimen, /p peranal excision on 2018-03-25, no residual tumor seen, but re-biopsy showed residual tumor on 2018-04-17, re-biopsy on 2018-07-02 still residual tumor
  2.Traumatic L1 compression fracture about 15 years ago w/o further management
  3.Eclipsy attack since 15 y/o under medication, last attack 2017-01

  During the CRS OPD, surgical intervention was suggested due to persistent positive result of his rectal cancer, though the patient hesitate for a long time. He finally accept surgery this time. Therefore, the patient was admitted for schedule surgical intervention.

體檢發現

- 1081119 No side effect
  - General appearance :
    - easy looking , conscious clear , without cardiopulmonary distress
    - Vital signs自動帶入date 體溫(BT) 脈搏(P) 呼吸(R) 血壓(BP) 身高(BH) 體重(BW) BMI
      20200109 36.4℃ 73次/分 18次/分 154/101mmHg 161.4cm 59Kg 22.6
  - HEENT :
    - Eyes: no anemic conjunctiva, no icteric sclera, normal EOM
    - ENT: no otorrhea, no rhinorrhea, no erythematous mucosa, no injected tonsil, no deformity
  - Neck: supple, freely movable, trachea midline,no deformity, no engorged jugular vein , no palpable thyroid or lymph node enlargement
  - Chest:
    - Inspection: Grossly normal, symmetric and fully expansion, no accessory muscle used, no operation scar, no spider angiomata, no gynecomastia
    - Palpation: no palpable mass or nodule, no subcutaneous emphysema, no tenderness
    - Ausculation: clear breathing sound
  - Heart:
    - Percussion: normal heart size
    - Ausculation: regular heart beat, normal S1, S2, no gallop, no murmur
  - Abdomen:
    - Inspection: in
      - flat contour, symetrically, no scar, no stria, no dilated veins, no rashes
    - Ausculation: normoactive bowel sound, no abdominal bruits
    - palpation: soft, no muscle guarding or rigidity, no local tenderness, no rebounding pain, no palpable mass, no palpable pulsation
    - Percussion: no shifting dulllness, no hepatomegaly, no splenomagaly
  -

Peripheral pulsation :	CA	BA	RA	FA	PA	PTA	DPA
Right side :	++	++	++	++	++	++	++/+++
Left side :	++	++	++	++	++	++	++/+++

  - Genito-anus: no tenderness or palpable mass lesion or rectal shelf, normal external genitalia, normal anal tone, brown stool

CRS CONDITION

-
  - Lung

手術日期及方法

- 手術日期及方法20200110 Low anterior resection with colo-anal anastomosis Ileostomy Laparoscopy

住院治療經過

- After the admission, the patient recieved pre op survey which showed no contraindication for surgery. The patient received Laparoscopic low anterior resection colo-anal anastomosis, end to end anastomosis, loop ileostomy on 2020/01/10. He tolerated it well and recovered smoothly. Due to his relative stable condition, the patient was discharged and arranged OPD follow up.

合併症

- nil

一般檢查

- 累積報告 :

DATE	TIME	ALB	TP	BIL.T	BIL.D	ALKP	AST	ALT	LDH	CHOL	TG	HDL-C	LDL	UA	NH3	AMY
1090110	1654	4.0	6.8			167	30	21		244	90		163	4.6		
1090110	1957	3.7		0.4				19								
1090113	0935			0.55			25	13								

1090114	1057	3.3														
1090116	0938			0.4			23	11								

DATE	TIME	WBC	RBC	HGB	HCT	MCV	PLT	BLST	NEUT	BAND	SEG	LYM	MON	EOS	BAS
1090110	1653	2790	3.53	12.0	36.5				52.0			38.0	7.5	1.8	0.7
1090110	1957	11390		11.8	36.5		199		84.1			12.5	3.2	0.0	0.2
1090113	0851	12290	4.11	13.6	42.1	102.4	182		79.2			15.3	5.1	0.2	0.2
1090116	0944	6480	3.69	12.3	37.0	100.3	200		68.6			22.7	5.4	2.8	0.5

DATE	TIME	NA	K	CL	CA	BUN	CREAT	Mg	P	CRP	ESR	PCT	LIPASE	LACTATE
1090110	1654					8	0.63			0.201				
1090110	1957	142	4.5		8.2	9	0.65							
1090113	0935	136	3.8			8	0.53							
1090113	1153													10.2
1090116	0933													9.7
1090116	0938	136	3.7			8	0.62							

DATE	TIME	GLUCOSE	PAB	TRF	ZN	CU	KETONE	CK	CKMB	PT-P	PT-C	APTT-P	APTT-C	TRO-T
1090110	1654	96												

- 放射線報告 :**檢驗項目 :SONO,ABDOMEN 報告日期 :1090117**

主　訴：
Result:
Finding:
1. Slight coarsening of liver parenchyma
2. A 0.9cm cyst at right lobe of liver
3. Several small gall bladder polyps are noted, up to 0.4cm. Presence of comet-tail artifacts of gall bladder, in favor of adenomyomatosis.
4. Several renal cysts in left kidney, up to 2.9cm.
5. Right kidney is 11.3cm. Left kidney is 11.2cm. No hydronephrosis.
6. Pancreas and spleen are not unusual.

Impression:
1. A hepatic cyst.
2. Gall bladder polyps and adenomyomatosis of gall bladder.
3. Left renal cysts.

檢驗項目 :CHEST AP VIEW (PORTABLE) 報告日期 :1090113

主　訴：
Result:
Finding:
> The heart size is within normal limit.
> Mild infiltration over bilateral lower lung fields
> Subcutaneous emphysema over left chest wall
> Tortuosity of thoracic aorta with calcifications
> DJD change with spur formation and scoliosis of T-L spine
> Comminuted fracture and deformity of right shoulder joint
> Old fracture of right 4th rib
> On NG tube, chemoport, and EKG leads

Impression:
As above description.

檢驗項目 :CHEST PA VIEW 報告日期 :1090113

主　訴：
Result:
Finding:
1. Calcification in the tortuosity of thoracic aorta, no cardiomegaly.
2. Scoliosis and severe spondylosis of the thoracolumbar spine.
3. Rectal cancer, S/P chemo port insertion. No active lung lesion is noted.
4. Otherwise, no other significant finding.

Impression:
As above description.

檢驗項目 :CT, PELVIS 報告日期 :1081208

主　訴：
Result:
Finding:
Post IV contrast CT scan of the whole abdomen with 600cc oral contrast showed:
1. History of rectal cancer, s/p CCRT. For follow up.
2. Segmental wall thickening of rectosigmoid colon, colon cancer is compatible. Relative stable condition is considered as compared with previous CT on 2019/8/21.
3. A 0.6cm small low attenuation lesion at S8 of liver, in favor of small hepatic cyst.
4. Hypertrophic change of bilateral adrenal glands.
5. Several cysts in bil. kidneys, up to 2.5cm.
6. Pancreas, spleen and gall bladder are not unusual.
7. No significant retroperitoneal lymphadenopathy.
8. Bil. visible lower lungs are clear.
9. DJD change of T and L spine.

Impression:
Compatible with rectosigmoid colon cancer, s/p CCRT, with stable abdominal condition as compared with previous CT on 2019/8/21.

特殊檢查

病理報告

其它

出院時情況

- 3.改門診治療

出院指示

藥名	劑量	劑量單位	用法	頻次	數量	數量單位	現況	天數	單位
(口)Takepron OD tab 30mg	1	TAB	PO	QDAC	16	TAB	TH	16	天
Keppra tab 500mg	1	TAB	PO	BID	32	TAB	TH	16	天
Tegretol CR-200mg	2	TAB	PO	BID	64	TAB	TH	16	天
Anxiedin tab 0.5mg	1	TAB	PO	QN	16	TAB	TH	16	天
Ultracet tab	1	TAB	PO	QID	64	TAB	TH	16	天

出院計劃

主治醫師_2062F 蔣鋅帆________ / 住院醫師_2328K 邱俊凱________

正式摘要(電子簽章：**000000184299669)**

W55 / W55

doctorRank:B
